# [Re]moving Bodies – A Shared Diminished Reality Installation for Exploring Relational Movement

**DOI:** 10.3389/fpsyg.2021.539596

**Published:** 2021-11-26

**Authors:** Julien Laroche, Loup Vuarnesson, Alexandra Endaltseva, Joseph Dumit, Asaf Bachrach

**Affiliations:** ^1^Center for Translational Neurophysiology of Speech and Communication, Italian Institute of Technology, Ferrara, Italy; ^2^UMR 7023 Structures Formelles du Langage, St Denis, France; ^3^École Nationale Supérieure des Arts Décoratifs, Paris, France; ^4^Emotic, Nantes, France; ^5^UMR 5044, Centre d’Etude et de Recherche Travail, Organisation, Pouvoir (CERTOP), CNRS, Toulouse, France; ^6^INSERM U1276, Centre d’Étude des Mouvements Sociaux (CEMS), EHESS, Paris, France; ^7^Science and Technology Studies, University of California, Davis, Davis, CA, United States

**Keywords:** virtual reality, interpersonal coordination, phenomenology, joint action, social interaction, shared experiences

## Abstract

In this article we explore an epistemic approach we name dis/embodiment and introduce “Articulations,” an interdisciplinary project bringing together Virtual Reality (VR) designers, cognitive scientists, dancers, anthropologists, and human–machine interaction specialists. According to Erin Manning, our sense of self and other emerges from processes of bodying and relational movement (becoming oneself by moving in relation with the world). The aim of the project is to exploit the potential of multi-person VR in order to explore the intersubjective dynamics of relational movement and bodying, and to do so with scientific, artistic and therapeutic purposes in mind. To achieve this bridge, we bring up a novel paradigm we name “Shared Diminished Reality”. It consists in using minimalist representation to instantiate users’ bodies in the virtual space. Instead of using humanoid avatars or full body skeletons, we reduce the representation of the moving bodies to three spheres whose trajectories reflect the tracking of the head and the two wrists. This “diminished”virtual rendition of the body-in-movement, we call dis/embodiment. It provides a simple but clear experience of one’s own responsive movement in relation to the world and other bodies. It also allows for subtle manipulations of bodies’ perceptual and cross-perceptual feedback and simplifies the tracking and the analysis of movements. After having introduced the epistemic framework, the basic architecture, and the empirical method informing the installation, we present and discuss, as a proof-of-concept, some data collected in a situated experiment at a science-art event. We investigate motion patterns observed in different experimental conditions (in which participants either could or could not see the representation of their own hands in the virtual space) and their relation with subjective reports collected. We conclude with reflection on further possibilities of our installation in exploring bodying and relational movement.

## Introduction

How can we use Virtual Reality (VR) to better study the role of the movement interactions in our experience of self and other? To approach this question, we propose an epistemic framework, “dis/embodiment,” where the body is not taken for granted as physical but is actively constituted and experienced through the relational movement between “the self”and “the other.” After explaining this approach, we demonstrate the benefit of using VR as a dis/embodiment technique. We then introduce the concept Shared Diminished Reality (SDR) to describe a minimalist multi-user VR design. To demonstrate the potential of SDR to support dis/embodiment research, we describe “Articulations,” an interdisciplinary project at the border of philosophy, art installation and empirical cognitive sciences. We present an analysis of the data collected at the event “Moving Humans: with CREATE” at the Tate Modern, a proof-of-concept situated experiment within a public museum event. Finally, we discuss how “Articulations”and its design contributes to the philosophical and scientific questions about relational movement and bodying.

### From the Experiential Body to Bodying Together Through Relational Movements

According to Erin Manning (2013), bodies do not pre-exist as objects within a pre-existing environment (or world); bodies as subjects co-become with the world they move in. Philosopher Brian Massumi builds on this: “*movement moves individuation, and in the process makes that ultimate chunk we call our body an event requiring a verb—bodying*” (Manning, 2013, p. xxiii). Hence moving is at the heart of bodying and worlding, and it is through relational movement that the subject-world is constituted. An epistemic shift from the “movement of bodies”to “bodying”enriches our understanding of movement with emotional and ethico-affective dimensions: how emotions, motions, and consciousness feed into each other (Foolen et al., 2012) in the ongoing formation of what we call “our body.” It invites the study of body-in-movement as “*the knowing self [which] is never finished, whole, simply there and original* (…) *able to join with another, to see together without claiming to be another*” (Haraway, 1988, p. 586).

This relational perspective on movement relates to Goffman’s (1978) research into how selves are constructed through their performative interactions with objects and persons, and to Blumer’s (1969) description of how meanings of self and others are made in interaction. We extend Goffman’s (1978) and Blumer’s (1969) constructivist approaches, inviting an understanding of the body as an emergent relational happening. We draw our epistemic inspiration from Donna Haraway’s notion of “apparatus of bodily production”(Haraway, 1991) which points to how things and bodies—material bodies, semiotic devices, and discursive tools—are always in relational becoming. We also build upon the enactive approach that situates the emergence of embodied experiences in a relational sensorimotor loop between an agent and the world she engages with (Varela et al., 1991) together with others (De Jaegher and Di Paolo, 2007; Fuchs and De Jaegher, 2009).

A number of psychologists and philosophers have long argued that interactions with others in particular take an important place in the constitution of the self and its behavior (Merleau-Ponty, 1960; Vygotsky, 1975; Penuel and Wertsch, 1995; Kyselo, 2014), blurring and making malleable the boundaries between the self and other (e.g.,Mead, 1934). The inherent circularity between the movements of self and other has been described as a self-organized, dynamical process by the enactive approach, which has discussed how movements of interacting agents get coordinated within the interaction process itself (De Jaegher and Di Paolo, 2007; Dumas et al., 2014; Laroche et al., 2014). Manning (2009) names such interpersonal (or, as she puts it, trans-subjective) dynamics “relational movement.” According to Manning (2009), by moving together, we are bodying each other. In that sense, the experience of our (and others’) bodies, or “bodyings,” emerges from relational movements, both during development (Stern, 1985) and during the micro-genesis of our instantaneous interactions (Manning, 2013; Sheets-Johnstone, 1999).

This body we experience as relation can potentially be the locus of a variety of disorders related to disembodiment as well as “relational”syndromes (for schizophrenia, see Varlet et al., 2012; Kyselo, 2016; (for autism, see for example De Jaegher, 2013; Koch et al., 2015; Fitzpatrick et al., 2016; Peper et al., 2016). We speculate that if bodying emerges through relational movement then abilities and disabilities, cognitive and body norms and deviations can be approached through the understanding of the qualities and properties of relational movement. In other words, what has been perceived as a “faulty”or “broken”body might be approached as a relational mismatch. The relational perspective in disability studies understands dis/ability as a relationship between an impairment and environment (Moser, 2006; Shakespeare, 2014; Goodley, 2017). While we do not directly address the topic of dis/ability in this paper, this relational perspective has served us as an inspiration for how the inquiry of bodying and relational movement could be enriched and taken further.

Erin Manning’s articulation of “bodying”offers an opportunity for alternative epistemic approaches to the bodies that differ or deviate from medical or heteronormative “norms”: bodies (and not only human bodies) which are multiple and compose with each other, bodies which escape fixation. We therefore inquire about ways of studying bodies without taking them for granted: how to stick to relationality and movement in the scientific interrogations of the body? “Not taking for granted”in scientific interrogations of (not only) bodies is what we learn from feminist embodiment approaches (e.g., Haraway, 1988) to compose our conceptual frame. This does not mean a plunge into constructivism. Rather, this move is somewhat close to Latour’s (2010)“compositionist manifesto”—a refusal to fall into universalism or relativism in knowing the world and a commitment to rethink the modes of being/acting in the world of scientific facts. For Latour (2010, drawing on Whitehead), bifurcation between the objective and subjective, appearances and reality is a slippery slope in debates about nature and its futures. Meanwhile, compositionism, putting diverse parts together without threatening their diversity, is “*a search for the Common*” with “*caution and precaution*” (ibid, pp. 487–488; also in Endaltseva and Jerak-Zuiderent, 2021). When it comes to bodies, Latour’s claim that “*the task of searching for universality but without believing that this universality is already there, waiting to be unveiled and discovered*” (Latour, 2010, p.474) might start from removing bodies as we know them. This is not to neglect the traces of socio-cultural and hierarchical compositions we embody but to take in that “*what is to be composed may, at any point, be decomposed*” (Latour, 2010, p.474). We propose that bodying and disembodiment are conceptual sisters—moving each other into action, composing and decomposing what is known with, about, and from the body which is always in relational movement with people and things at place. Manning (2009) argues that “*we move not to populate space, not to extend it or to embody it, but to create it*” (Manning, 2009, p.12). Thinking together with Manning and Latour we endeavor to operationalize an epistemic and methodological configuration that does not take bodies for granted, harnessing the movement between disembodiment and bodying as part of the apparatus. We name this conceptual turn “dis/embodiment”: by suspending the habit we call “our body,” we can provide a novel window (from first and third person perspectives) into the diversity of bodying experiences.

In the next section, we show the benefit of using VR as a technique to approach dis/embodiment, then we propose a materialization of such a methodology in a device called “Articulations”using multi-person VR along minimalist design, a technique we call SDR.

### Virtual Reality as a Tool for Dis/embodiment

We propose that VR is a vehicle to approach dis/embodiment. By providing highly realistic and immersive stimuli, VR tools can easily fool the cognitive system and therefore alter the perception of the world and the self. This allows for paradigms that are “*valid and highly ecological without compromising experimental control*” (Loomis et al., 1999). Different kinds and degrees of dis/embodied experiences can then be designed and tested. Moreover, VR tracking systems allow for precise measures of the users’ kinematics, without requiring an additional motion capture system. Practically speaking, VR setups are now easy to transport and to install in various venues. Therefore, such setups are easily reproducible “in the field”in experiments with identical parameters and sensorial conditions, as well as quickly customizable to fit the actual environmental configuration (dimensions, length of experimentation, number of participants). What follows is a brief review of empirical research on/in VR which we introduce as a demonstration of its potential for dis/embodiment studies.

The question of the body and its appearance in the virtual world is one of the most important topics in the design of VR environments (Murray and Sixsmith, 1999). Indeed, the constitution of our body’s experience seems to be as complex and subject to multiple factors in the virtual world as it is in “normal”reality. The “*Proteus effect*” (Yee and Bailenson, 2007) describes how manipulating the features of a person’s avatar modulates her behavior and her affect, with effects lasting even after the immersion. In other words, VR can induce new perceptions of one’s own emotional and physical capacity. For instance, people behave more confidently with taller avatars (Yee and Bailenson, 2007; Yee et al., 2009) and they report more negative and aggressive thoughts if their avatars are dressed in black or in Ku Klux Klan outfits (Peña et al., 2009). In general, differential embodiment effectively modulates and changes the way we think, feel and move in socially and culturally patterned ways (Banakou et al., 2013; Kilteni et al., 2013; Peck et al., 2013).

The alteration of a body’s position in the virtual space can also create a physical discomfort which subjects are typically trying to compensate for, although their “real” body position was actually comfortable in the first place (De la Peña et al., 2010). The modulation of behavior and affect by bodilyrelated experiences also happens with less anthropomorphic avatars. For instance, robot-like avatars tend to produce a certain feeling of security when facing a dangerous situation (Lugrin et al., 2016). The same goes for the shape taken by the virtual other: the mere choice of avatar skin color has an effect on the willingness of a group of individuals to collaborate (Wallace and Maryott, 2009). In contrast, a balanced combination between visual similarity among members and self-identification enhances social attraction between them, as well as their motivation to contribute to a group task and the task performance itself (Van Der Land et al., 2015). Importantly for our purpose here the feeling of social presence isn’t correlated to the anthropomorphic realism of avatars (Nowak and Biocca, 2003).

In sum, with its capacity to modify, transform or compose with the appearance of “real world”percepts (related to ourselves, others, and the environment), VR lets us reconstruct and discover new elements of our self as we perform our identity through self-exploration and role-playing (Turkle, 1995; Taylor, 2006). It allows for experiencing and experimenting with the links between bodying and relational movement in a controlled and replicable manner.

### Shared Diminished Reality: Re-moving Bodies

To investigate relational movement and bodying through dis/embodiment, we have developed a multi-person VR installation where the immersed participants are co-present via minimalist dis/embodiment. We call this minimalist paradigm SDR, as we retain basic elements of interactive behaviors yet limit the range of possible actions and the amount of perceivable information. This allows participants to focus their attention on the interaction process and permits us to capture and to analyze body motion more easily and readily.

In order to design a VR environment that challenges what is taken for granted about the body, we chose to stay away from anthropomorphic avatars and to invite users to experience bodying without most of its usual properties. Indeed, extensive research has demonstrated the ease with which we can still perceive human biological motion (Johansson, 1973), animateness (Heider and Simmel, 1944), emotion (Dittrich et al., 1996), and interactional behaviors (Froese et al., 2014a) from a very reduced set of information consisting of a few moving points. Following a similar logic, we argue for the simplification of the virtual body’s appearance to a very minimal form (three spheres in our case). Since both our dis/embodiment approach and our interest in relational movement eschews anthropomorphic realism, we avoid the conundrum of the “neutral.” In other words, we do not need to create de-sexualized/de-racialized avatars or make arbitrary or normative choices concerning body shape and appearance. In order to avoid confounding or distracting factors (e.g., independent movement, additional objects, attention-grabbing details), and facilitate real time data streaming, data recording and modeling of interactional behaviors, we also chose to reduce to a minimum the virtual environment itself.

Engaging the main theme of this special issue, SDR allows for experimentally controlled dis/embodiment. For example, we can manipulate the appearance, position, and dynamics of the points representing the avatar, and we can play with the amount of information provided by the environment and the interacting avatars. We can thus modulate and favor the participants’ behavior, affect and interaction dynamics that are of direct interest to us. This way, SDR enables us to investigate the possibility for human beings to interact, being deprived of most of the usual communication means such as voice, body posture, facial expression, hand gestures. The underlying assumption here is that getting rid of these sociocultural and linguistic modes of communication would help to highlight (for the experimenters and participants) the dynamics of relational movement that constitute our collective bodying.

What follows in the next part is a detailed presentation of our project “Articulations,” which implements a specific instantiation of SDR for the study of bodying and dis/embodiment. We first introduce the project and the techno-scientific framework behind the design and the construction of this device. Then, we describe its modus operandi through a presentation of an art-science public experimentation that took place in June 2019 on the occasion of an science-and-art event at the Tate Modern in London.

### Articulations : A Design Project and an Installation

Starting in January 2019, the project “Articulations”has brought together VR designers, cognitive scientists, dancers, anthropologists, and human–machine interaction specialists. The aim was to develop a methodological and technical dis/embodiment installation (“Articulations”) that (1) augments and modulates users’ sensitivity to the relational experiences of dis/embodiment and bodying and (2) permits scientists to experiment on, observe and interrogate the trans-subjective processes that underlie dis/embodiment and bodying. The central idea has been to offer a collaborative experience by immersing two users, using portable headsets, in an SDR environment, dis/embodying each of them as a set of three floating spheres whose animation is derived from the movement of their head and two hands. Moving between different disciplines and perspectives on the body, the “Articulations”multidisciplinary team engaged in a process of participatory and immersive design. As a result, relationality and bodily lived experiences that the installation aims at highlighting were core parts of its design process itself.

The development of the first “Articulations”prototype took 3 months, after which a group of artists, scientists, and philosophers was invited to spend 3 days in a brainstorming residency with the project team. During the residency, each participant explored the SDR prototype with various partners and also observed others exploring it, thereby soliciting insights from multiple perspectives. We spent over 10 h in facilitated meetings sharing our own experiences and discussing the potentials and limits of the installation in order to improve its design. Following the residency, we organized a first public event with local volunteers (recruited through email and social networks announcements). We developed a quantitative and open-ended questionnaire addressing participants’ lived experiences of the device itself, of their own body feelings as well as their relational experiences. We also designed a series of questions to be given to participants in post-experience discussions. The experiences shared by participants informed the next cycle of technical modifications and the creation of a general experimental protocol.

**FIGURE 1:**
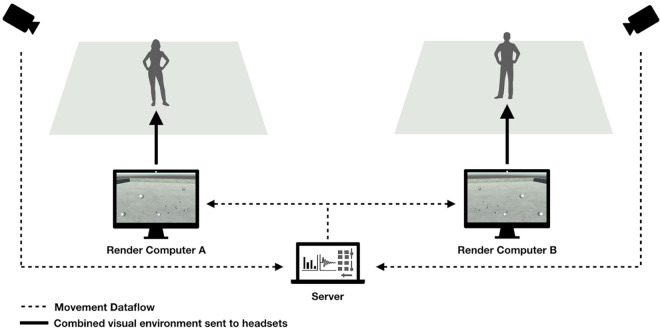
Graphic illustration of the Articulations setup: the two participants are in separate squares but share the same virtual space. HTC Vive lighthouses are installed around the movement space and provide the headsets with localization information. The data is then processed by two dedicated computers, connected via a server program installed on a third computer, that render the virtual scene.

**FIGURE 2:**
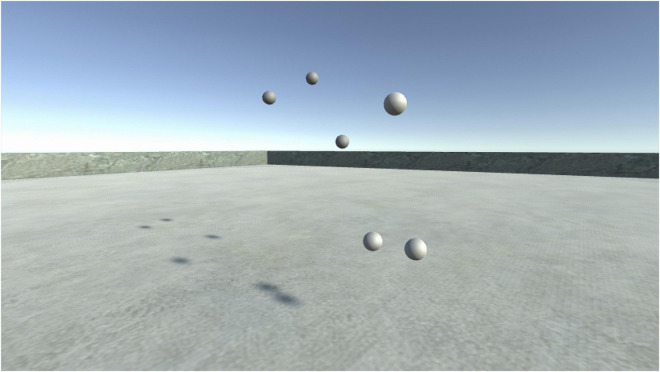
Visualization of the virtual scenery from a third person point of view. We can see the two participants each symbolized as three spheres in the virtual space.

In this protocol, both users have been placed in separate physical squares in order to prevent collisions (Figure 1) yet participate in the same space in the virtual environment where they can move, walk and interact in real-time. The virtual space itself has been made exceedingly simple: users were placed on an empty plane, under a luminous and wide blue sky (see Figure 2).

Concerning the interaction design, we conceptually draw on Manning’s proposition that dance practice orients our attention to the movement of bodying: “*There are an infinity of ways of touching on the more-than that is movement-moving. Dance is one example. What dance gives us are techniques for distilling from the weave of total movement a quality that composes a bodying in motion*” (2013, p. 14). To observe general and spontaneous aspects of relational movements under various conditions of dis/embodiment, the protocol thus invites users to explore the environment (which includes their partner) freely, in a non-goal oriented, non-constrained manner. This is what one would do in Collective Dance Improvisation (CDI), where the activity of the dancers is based on the qualities they experience in moving together (Himberg et al., 2018). Conceptually, dancing is the way we learn to body, that is, to experience and interact with a world.

## A Situated Experiment at the Tate Modern

In June 2019, we were invited to present our device during the workshop “Moving Humans” at Tate Modern museum (London, United Kingdom) organized in collaboration with CREATE (University of London). The event was free and open to the public. For the purpose of this event, we created a specific protocol that allowed us to combine an artistic and playful experience with a scientific agenda.

### Situated Experimentation

Our interest in introducing a SDR installation into the hybrid art/science event is related to a growing trend of scientific research based on, or targeting, artistic practices (Skov et al., 2018). However, it has a distinctively different logic, inspired by Latour’s (2010)“compositionist”search for new ways of acting in the world of scientific facts. We draw upon a specific genre of “acting science”which Latour has developed throughout his collaborative “thought exhibitions”such as Making Things Public: Atmospheres of Democracy or Critical Zones to propose an extension of a “method exhibition”or “data collection exhibition.” In other words, we mix data collections, reflection on the data collection, and the demonstration/explication of the data collection for finding a mode of collaborative design *in situ*. Therefore, the protocol which follows does not fit nicely into the category of a lab-experiment, nor is it simply data collection in the field or a scientific observation of an artistic process/event. The protocol is linked with a particular event where the scientists, artists and public all have their respective “agendas”which might coincide or not (unlike the concept of a subject of a psychology experiment that “serves”the agenda of the experimenter). The protocol integrates multiple contingencies and specificities that cannot be meaningfully replicated, given that it is exactly these singularities that make the event a “happening.” For the scientists in the project, the ambition was to be able to collect quantitative data and to be able to observe the effect of slight modification of the experience on the (relational) movement of participants, relating these differences to differences in the lived experience of the same participants. Given the singularity of the event, and since our epistemic approach does want to embrace and not abstract away from real-world complexity, the criteria assumed behind standard statistical/analytical methods are often not met. However, as part of the process of developing custom analysis tools, we chose to apply standard methodology as an attempt to provide connections between this epistemological approach and more canonical experimental results in the literature.

The section below presents the resulting art-science happening prototype, and some ensuing observations. It can be thought of as a report of proof of concept(s) departing from dis/embodiment as an epistemic framework. An epistemic stance of dis/embodiment, we recall, allows a compositionist approach (Latour, 2010) to the body in scientific research (and not only) where the body and its physical composition are not taken for granted. This suggests a possibility to study bodying experiences as they emerge relationally from movement. The event described below works with this suggestion, hypothesizing that a specific form of VR, we called SDR, provides, first, a material framework for dis/embodiment as a concept, and, second, a methodological opportunity to experiment with it. We anticipate that our prototype of SDR, an installation called “Articulations,” would allow linking experiential and qualitative investigations of bodying with experimental and quantitative examination of relational movement. Finally, we anticipate a proof of concept on the feasibility of situated art-science experimentation as we present some meaningful data from an installation within the museum, where visitors, fellow exhibitors, and casual passers-by become experiment participants and observers.

### Design

A common feature of psychology experiments and (durational) art installations is the use of minimal contrasts (Bateson’s “differences that make a difference,”Bateson, 1979). Scientists and artists often introduce subtle changes into an ongoing “event.” For the scientist this is a way of disentangling the cognitive correlates of a specific parameter (e.g., perceptual changes). For the artist, minimal differences heighten the aesthetic tension of the work (Massumi, 2015). For the Tate, we chose to produce a 9 min continuous “scenario”within which we introduced, every 3 min, a more or less small change (changing the “condition,” in experimental parlance, to allow for contrasts to emerge). We developed two such scenarios, each exploring a slightly different set of questions: the “Transformed Bodies”scenario, which involved changes in avatars’ appearance, and the “Mirror”scenario, which involved interacting in presence of a mirror.

In the first scenario (“Transformed Bodies”), the avatars’ appearances were slightly different in each condition. In contrast to a baseline situation (where each avatar is represented by spheres corresponding the head and both the hands), a situation was introduced where the spheres representing participants’ own hands disappeared (but could still be perceived by the partner); in another one the spheres were displaced, giving the impression of either a stretch or a compression of the arm’s length. We expected that eliminating visual feedback of one’s own moving body (i.e., of one’s own hands) would shift the attention toward the other and the intersubjective space, since the sensorimotor coupling with the other’s movements would be the locus of feedback of one’s own movements (“perceiving myself through the consequences of my movements on yours”). We hypothesized that this shift would result in enhancing the coordination of movement patterns between the participants. In contrast, we expected that the changes in the perceived size of arms would decrease the ability to coordinate and their associated feelings.

In the second scenario (“Mirror”), we proposed to gauge the changes in behavior occasioned by the perception of a full representation of oneself and the other (all six spheres on the same plane), through a mirror that appeared in the environment. We expected that it would encourage an increase in collaborative movements in order to form shapes together. Furthermore, we wanted to explore the phenomenal and interactional consequences of reducing or adding color cues for self-identification. Specifically, we wondered if an increase in self-other differentiation would lead to an increase or a decrease in experiential and/or behavioral intersubjective coupling. Due to the technical problems, the data of only 6 dyads could be analyzed for this scenario. For this reason, we concentrate this report of proof-of-concept on the “Transformed Bodies”scenario, for which 10 dyads could be analyzed.

### Participation

Due to the particularity of the venue, the issue of participation played a singular role in our experiment. In the context of a museum exhibition, introduction of a strict laboratory-like research protocol is not possible. The first challenge is the flow of participants. In a museum, people move and wander freely, governed by their own curiosity. We chose to work with this open ended flow, rather than control it. We invited the audience to stop and watch the running experiments while a screen was showing what was being rendered in the virtual environment. As a consequence, some participants were aware of the idea behind the experiment before doing it, while others were not. Some knew each other and insisted on doing the experiment together while others were partnered randomly. This diversity of cases and contexts had an impact on the collected data. In a certain manner, the participants co-built the experiment with us. We endorse this particularity as part of the logic of the design of “Articulations”: the installation is participatory and invites suggestions for co-construction, insight and improvement. We consider the Tate event, therefore, a unique chance to see our experimental configuration go outside our expectations, making the scientific method itself a part of the experimentation.

In total, 46 participants volunteered for the experiments. 23 dyads were formed based on the order of arrival of participants and were randomly assigned to one of the two experimental scenarios (“Mirror”and “Transformed Bodies”). Due to the technical problems, the data of only 10 dyads (20 participants) could be analyzed in the “Transformed Bodies”(Age: 30.4 ± 11.4), and only for the baseline condition and the condition where the visual feedback of one’s own hands was suppressed. The participants of half of the analyzed dyads knew each other well. Only three participants had a very regular dance practice and only one had a regular experience with VR.

### Materials

The shared environment was created through three instances of the same custom created Unity program: Two Vive Pro headsets were connected each, via a Vive wireless adapter, to a client-instance running on a dedicated computer rendering the 3D environments at 60 frames per second. The third instance of the program was the server, gathering (and recording) the positional data sent by both headsets and hand trackers and sending them back to the connected clients. The server also acted as a remote controller of the two clients, triggering visual changes and events at specific times, manually or automatically. The VR headset, a wireless Vive Pro, offered a 110° field of view (approximately 90° per eye), with two 1,440 × 1,600 pixels displays. Coupled with two Vive Trackers strapped to the users’ wrists, this system allowed us to track and record hand and head movements in a three dimensional space (60 frames per second) as well as the voice during a specific phase of the experience.

### Procedure

The two participants were placed in two adjacent (separate but with no occlusion) physical spaces (approximately 4 m square each). Once fitted with the headset, participants discovered an “empty”world with only two visible objects (two spheres) corresponding to the location of the sensors located on their hands (in the article we often refer to these sensors/spheres as “hands”). They were invited to walk around the virtual space and explore its boundaries. After a short while, each participant was able to see three additional mobile spheres corresponding to the hands and head of their partner. The participants were not explicitly told about the mapping of spheres to body parts.

**FIGURE 3:**
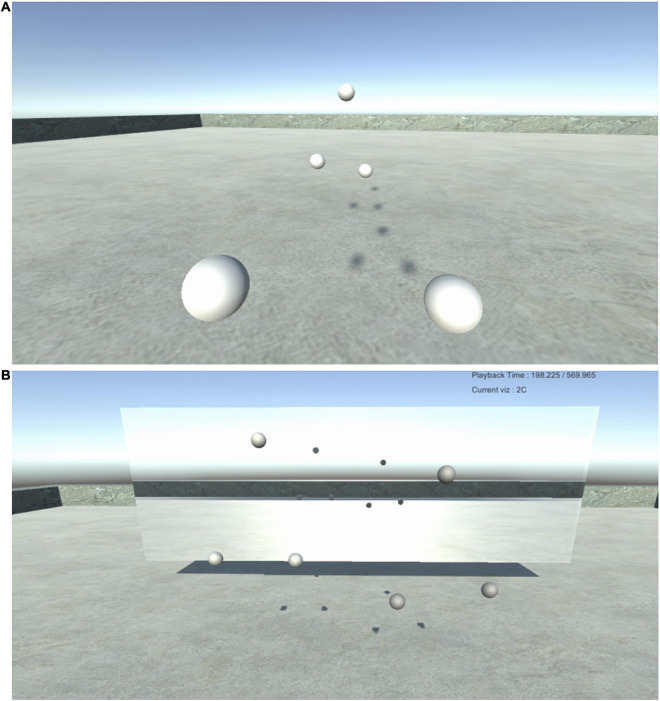
**(A)** Baseline condition: Visualization of the scenery from a first person point of view. The two closest spheres correspond to the participant’s hands. The three spheres at the back of the image correspond to the position of the head and two hands of their partner. Shadows of the floating objects are visible on the ground. **(B)** Mirror scenario: Visualization from a third person point of view. Here both participants are standing side to side in front of their own reflection.

The experimental protocol lasted 9 min and was divided into three“blocks”of 3 min, each corresponding to a different experimental condition and presented in a randomized order, without any interruption between them. In the “Transformed Bodies”experiment, one block served as a baseline, where both avatars were present, each consisting of three spheres of same size and color (Figure 3). In another condition (NO-HAND), participants did not see the spheres corresponding to their own hands but each of them could see the spheres representing their partner’s hands. In the RESIZE condition, the spheres representing the hands were made either more or less distant from the virtual body than they were in the baseline giving the impression of a stretching or shortening of the virtual “arm.”

At the end of the experience, the partner’s avatar would disappear, and each participant would find themselves once again alone in the virtual environment, seeing only their own hands. A pre-recorded voice in the headset invited participants to express out loud their experience for about 1 min. The headset microphone allowed us to record their voice while they stayed immersed. After speaking, and once the tracking equipment has been taken off, participants filled in a questionnaire about their lived experience inside the virtual space. As the participants were finished with filling out the questionnaire, they were invited for an open-ended interview with one of the team members. An open-ended interview analysis is beyond the scope of this paper, however. The next section will elaborate on the logic of the questionnaire construction.

### Experiential Reports

The goal of the questionnaire was not only to obtain feedback on the device and the experiences it affords but also to check if lived experiences reported by participants could be related to the observable patterns of (individual or relational) bodily behaviors. It was composed of assertions (presented on a tablet) with which participants indicated their agreement on a seven-point Likert scale. We chose this approach (rather than an interrogative form) since it matched our linguistic sources (participants’ description of their experiences on earlier occasions) and simplified and homogenized the task for the participants. We were interested in scalar responses as this would allow us to correlate the experiential reports with the movement variables. If we asked different scalar questions (e.g.: “*To what extent did you enjoy the experience?”*, “*To what extent did you feel connected to your partner?*”), each question would require an ad-hoc mapping of the specific content of the question onto a scale. The strength of adherence to an assertion, however, can be modeled as a continuous effect which is independent of the content of the assertion and which (from our experience) simplifies the task for participants.

The choice and wording of assertions was grounded in reports collected from the users of the installation during the previous experimentations and collective retreats. Reviewing the answers to the questionnaires and the transcripts of the conversations from these earlier events, we identified statements that identified specific aspects of personal experience that appeared more than once. We extracted representative statements and those prototypical to the categories of the experience most relevant to our research interest (relational movement, bodying, dis/embodiment). Importantly, we formulated the questionnaire by staying as loyal as possible to the wording from the first-person experiences, reformulating them only when the language needed to be specified, clarified, or stylistically adjusted. Adhering to the verbal descriptions provided by the participants themselves was fundamental to keep away from our abstractions and expectations addressing the subjective experiences directly. This approach to building a questionnaire disciplines us to stay “compositionist”(Latour, 2010) with bodying, and not taking bodies as fixed objects.

Most of the questions addressed the general experience (e.g.,“*I would have liked the experience to continue much longer*,” “*The minimality of the setup made me surprisingly more curious and explorative*”). A second smaller set of questions targeted the experiences specifically felt during particular experimental conditions. In the analysis presented here we focus on the responses to the latter, in particular to the two questions that inquire about feelings experienced during the analyzed manipulation. Namely the elimination of one’s own “hands”: (a) “*The absence of my own spheres made me feel more closeness with my partner*”and (b) “*When I could not see my own body I found myself more interacting with my partner*”). We will report how the responses to these two questions related to observed patterns of bodily behavior.

### Data Processing, Measurements, and Analysis

**FIGURE 4:**
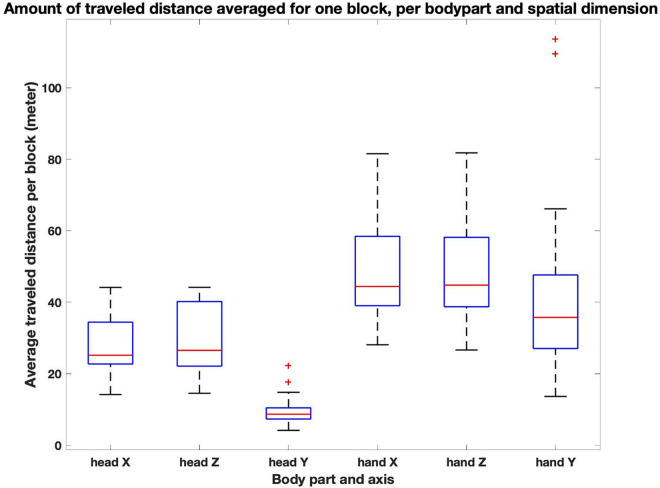
Average amount of distance traveled by block per body part and spatial dimension. In general, distances were quite similar in the X and Z dimension, which reflects the square nature of the virtual space. For the head, displacements along the vertical dimension were significantly smaller than along the horizontal ones (signed-rank Wilcoxon tests; *z* = 4.11, *p* < 0.0001). For the hands, displacements along the vertical dimension are also significantly smaller than along the horizontal ones (*z* = 2.74 and *z* = 2.71, *p* < 0.01 for both vertical vs horizontal comparisons) yet the respective amount of displacements have a comparable range.

In order to quantify how participants moved in the virtual space across conditions, we considered hands and head motion separately. What we mean here by “head”and “hands”are the markers provided by motion capture devices placed on the participants’ wrists and the headset. As can be seen in Figure 4, vertical head motion happened more sparsely and only intermittently, while participants’ hands explored all three dimensions equally. Though we did not collect torso motion data, we repeatedly observed that once immersed, participants mostly did not move the head as an independent effector. Rather, head movement was closely related to the overall motion of the participants trunk in the virtual space. Given these considerations, we took the head’s horizontal motion as a proxy for the (horizontal) displacements of the participants in the virtual space (X and Z). For the hands, we took into consideration the overall movement of both hands in the three-dimensional space (X, Y, Z). We first interpolated the original positional time series data at 100 Hz and lowpass filtered them with a second-order Butterworth filter with the cutoff frequency set to 5 Hz in order to eliminate noisy jitter. Then, as a first step in the exploration of the data and with the aim of developing more comprehensive non-linear analysis tools in the future, we computed three types of measurements from the resulting series.

Median Velocity (MV):we first obtained velocity time series by differencing the positional time series of each dimension. Next, each datapoint was squared to obtain unsigned velocity values. Then, we summed the time series of the different dimensions (X and Z for the head, X, Y and Z for both hands), and we computed the square root of each resulting datapoint in order to obtain time series of the overall velocity of the hands and the head, respectively. Finally, we extracted the median of each of the resulting head and hands time series. Thus, for each subject in each condition, and for both the hands and the head, we obtain a singular value reflecting the median velocity of movement.

Relative Head Velocity (RHV): we computed the real-time difference in velocity between partners’ head motion in the horizontal plane. To do so, we used the velocity time series described in (1) from both partners for each axis of the horizontal plane (X and Z), to compute a new time series for each axis representing the difference in velocity at every time point between partners. Next, each datapoint in the new time series was squared in order to obtain unsigned difference values. We then summed the time series of the X and Z axis and we computed the square root of each resulting datapoint in order to obtain a time series of the real-time absolute difference between head velocity in the horizontal plane. Finally, we extracted the median of the resulting time series, which provided us with an index of the median instantaneous relative velocity for each pair of participants in each condition.

**FIGURE 5:**
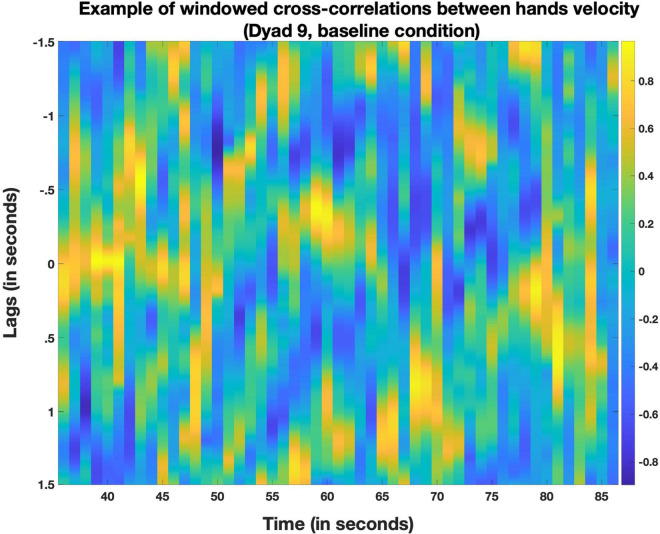
Example of windowed cross-correlations between hands velocity of two partners (dyad 9, baseline condition). Each window is 2 s long. The one second overlap between successive windows entails the creation of a new window at every second of the velocity time series. For every window, a correlation coefficient with the partner time series was computed at every lag between 0 and 5 s, in this example in both time directions (e.g.,correlations with what the partner did up to 5 s earlier as well as later). The resulting correlogram plots the computed coefficient for every window (x axis) at every lag (y axis). The color coding indicates the strength of that correlation coefficient, with cold, most blueish colors indicating strong negative correlation and hot, most yellowish colors indicating strong positive correlations. A positive correlation along the Y-axis 0 indicates synchronous coordination. A positive correlation at negative lags (in the upper part of the plot) indicates that a subject had similar movements to what her partner did earlier (a “follower”). A positive correlation at positive lags (lower part of the plot) indicates that the subject is “leading” (the partner will later make movements that are similar to those the subject makes in the current window). In this example, we can see that both partners first synchronously coordinated (high correlations around lag 0, from 35 to 45 s approximately) before entering a phase that can be described as turn-taking with partners shifting between leading and following.

Motion cross-correlation (MCC): we computed windowed cross-correlations between partner’s movements using the overall velocity time series described above and that we used to compute MV. We used moving windows of 2 s (with 1 s overlaps), which can correspond either to a slow movement or to a short sequence of coarticulated gestures. Cross-Correlations were computed over positive lags ranging from 0 to 5 s (by 10 ms overlaps). This allowed us to examine how the movement of each participant related to the present as well as the short-term past of the partner’s movement. The 5 s lag value was chosen because movement coordination can take many forms going from real-time synchronization to delayed imitation of a gesture, and can therefore be identifiable at a wide variety of lags (Tschacher et al., 2014; see also Figure 5). For this reason, within each window we extracted the peak correlation coefficient whatever was the lag at which it was found. This provided us with a time series indicating for each window the maximum coordination observable between the partner’s movement regardless of the delay between these time series. We then used the median of the Z-transformed time series of peak correlation coefficients to provide an index of coordination across time of each participant to his/her partner, in each condition, and for both the head and the hands.

As the distribution of data was not normal, we compared these three indices across conditions (baseline and NO-HAND) with Wilcoxon (signed-rank) tests. For the same reason, relating movement variables to subjective reports, we performed Spearman’s rank correlation between the responses to items of the questionnaire that targeted lived experiences during specific experimental conditions and kinematic indices.

### Quantitative Observations

#### Kinematic Measurements

**FIGURE 6:**
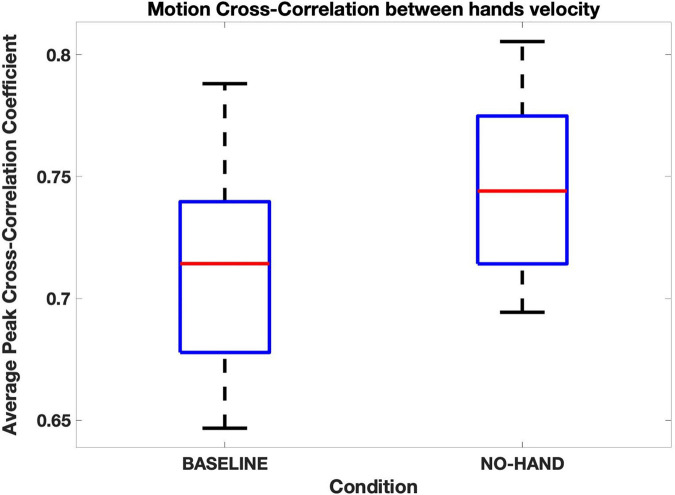
Motion cross-correlations between hands’ velocity. Average peak cross-correlation coefficients were significantly higher when participants couldn’t see their own hands (NO-HAND) than when they did (BASELINE).

The MCC between partners’ hands’ velocity was significantly higher in the NO-HAND condition than in the baseline (z = −2.87, *p* < 0.01; see Figure 6), but no significant difference was found for the head (see Table 1 for all paired comparisons). In other words, partners coordinated their hands (but not their head) more across time when they could not see their own. There was no significant difference in terms of MV or RHV between the baseline and the NO-HAND condition (see Table 1).

**TABLE 1:** Paired Wilcoxon sign-rank tests between BASELINE and NO-HAND conditions for the different kinematics variables.

**Variable**	**Paired Wilcoxon sign-rank test between conditions**
HEAD MCC	z = −1.08; *p* = 0.28
HANDS MCC	**z = −2.87; *p* = 0.004**
HEAD RHV	z = −0.97; *p* = 0.33
HEAD MV	z = −0.30; *p* = 0.77
HANDS MV	z = 0.37; *p* = 0.71

*Bold indicates significant comparison.*

#### Correlation Between Kinematic Measurements and Experiential Questionnaires

**FIGURE 7:**
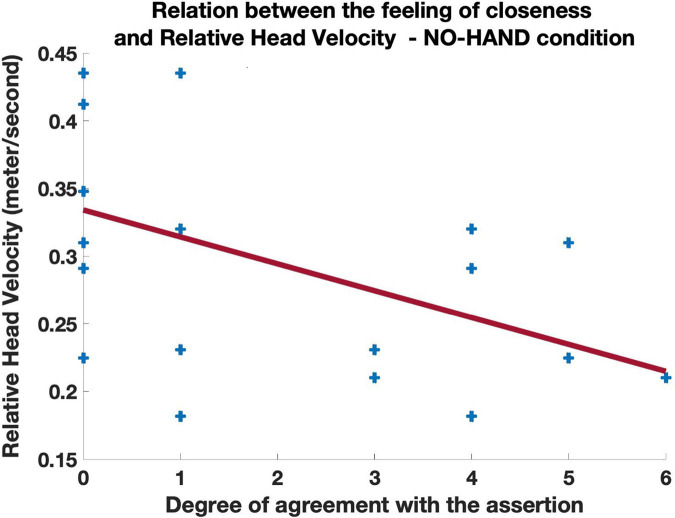
Scatterplot of the relation between the responses to the question “*The absence of my own spheres made me feel more closeness with my partner*” and the Relative Head Velocity. 0 meant total disagreement and 6 total agreement with the assertion, with values in between corresponding to more moderate responses.

**FIGURE 8:**
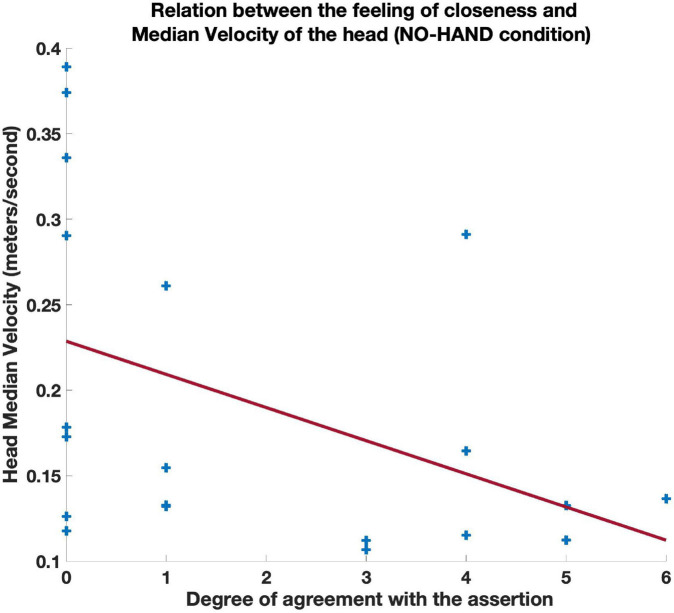
Scatterplot of the relation between the responses to the question “*The absence of my own spheres made me feel more closeness with my partner*” and the Median Velocity of horizontal head displacements. 0 meant total disagreement and 6 total agreement with the assertion, with values in between corresponding to more moderate responses.

We observed a negative correlation between the feeling of closeness induced by the absence of spheres (“*The absence of my own spheres made me feel more closeness with my partner*”) and RHV, but this correlation was significant only in the NO-HAND condition (r = −0.50, *p* < 0.05; see Figure 7). In other words, when their own hands were invisible, participants who moved in space in a more similar velocity, felt closer to each other. The responses to the same question correlated negatively with the participant’s head MV, but this correlation was also significant only in the NO-HAND condition (*r* = −0.46, *p* < 0.05; see Figure 8). Importantly, the feeling of closeness in that condition was not significantly connected to the head MV of the partner (r = −0.25, *p* = 0.28). These two results indicate that slower self-motion (but not that of the partner) was related to one’s feeling of closeness but only when participants could not see their own hands. All other correlation coefficients are reported in Table 2. It is important to note that these two movement variables (RHV and head MV) were correlated (r = 0.65, *p* < 0.01). This indicates that those who moved faster had more dissimilar head movements velocity than their partner. Given this collinearity, it is not possible to directly parse and gauge the respective and independent contribution of these two variables to the feeling of closeness.

**TABLE 2:** Spearman rank correlation between experiential reports and kinematic variables.

**Variable**	**Experimental condition**	**“*The absence of my own spheres made me feel more closeness with my partner”***	**“*When I could not see my own body I found myself more interacting with my partner*”**
HEAD MCC	BASELINE	r = 0; *p* = 1	r = −0.17; *p* = 0.47
	NO-HAND	r = −0.19; *p* = 0.42	r = 0.14; *p* = 0.55
HANDS MCC	BASELINE	r = 0.34; *p* = 0.15	r = 0.11; *p* = 0.55
	NO-HAND	r = 0.17; *p* = 0.48	r = 0.11; *p* = 0.64
HEAD RHV	BASELINE	r = −0.14; *p* = 0.55	r = −0.11; *p* = 0.65
	NO-HAND	r = −0.50; *p* = 0.02*	r = −0.33; *p* = 0.16
HEAD MV	BASELINE	r = −0.35; *p* = 0.13	r = −0.03; *p* = 0.90
	NO-HAND	r = −0.46; *p* = 0.04*	r = −0.35; *p* = 0.13
HANDS MV	BASELINE	r = −0.28; *p* = 0.23	r = −0.05; *p* = 0.84
	NO-HAND	r = −0.10; *p* = 0.68	r = −0.18; *p* = 0.46
HEAD MV of the partner	BASELINE	r = 0.23; *p* = 0.33	r = −0.03; *p* = 0.90
	NO-HAND	r = −0.25; *p* = 0.28	r = −0.06; *p* = 0.81
HANDS MV of the partner	BASELINE	r = −0.04; *p* = 0.86	r = −0.18; *p* = 0.44
	NO-HAND	r = −0.08; *p* = 0.74	r = 0.04; *p* = 0.87

Bold and * indicate significant comparison.

## Discussion

In this article we presented the “Articulations”installation for studying relational movement and its situated experimental testing at a science-art event. We first outlined an epistemic framework (dis/embodiment) for the scientific study of the body as an interactive process (bodying). We then introduced the design and operative particularities of SDR as a methodological device for such interrogation, and briefly speculated on its promises for relational approaches to disability. We followed with a description of the “Articulations”SDR installation, where the users are represented most minimalistically, through avatars consisting of three identical mobile spheres corresponding to their head and hands. We finally presented some preliminary results from a situated experimentation at the Tate modern. Our goals at this event were first to test the viability of a SDR set-up to produce and capture (quantitatively as well as qualitatively) bodying through relational movement and dis/embodiment. In addition, we aimed to evaluate the relationship (and our capability to apprehend it) between felt experience and quantitative measures.

We used the specific affordances of SDR to verify if certain manipulations of the visual feedback of one’s own movement (“Transformed Bodies”) would have significant and differential effects on behaviors, interactions, and lived experiences. We introduced the variations in the virtual environment in pseudo-randomized orders. In the “Transformed Bodies” experiment, the baseline situation was contrasted with one of increased dis/embodiment, where participants could only see the spheres representing their partner. Our expectation was that the disappearance of one’s own body (avatar) would draw one’s attention to the experience of bodying through relational movement and would favor (measurable) coordination with the partner’s movement as their reaction would embody or replace the missing feedback of the participant’s own body.

### Bodying Through Relational Movement

Behavioral results revealed that the velocity of hand movement was more coordinated across partners when the spheres representing participants’ own hands disappeared from their view, compared to the baseline situation where they could view their hands. In other words, the disappearance of one’s own spheres from one’s view, seeing only the other’s spheres, seems to have encouraged movement coupling in the dyad. We propose that this specific SDR manipulation provided for a situation where one’s own body(ing) was felt through the movement of the other. This dis/embodiment manipulation might have displaced participants’ attention toward the other and/or the dynamics of their sensorimotor relationship (in other words, the relationality of their movement). The strengthening of the reciprocal coupling between the participants might have been the consequence of an explicit attempt to communicate and/or a result of unintentional mutual entrainment. We thus interpret the increase in coupling as a reflex of bodying through relational movement. The body here is not a pre-set entity, detached from a pre-existing world; it is constantly composed as a bodying experience where the other becomes the main reference point. The “other”is active in the dis/embodiment experience since the perceptual trace of the consequences of one’s own movements in the world can be embodied only through the “other.” Thus, the visible body of the “other”co-becomes with the personal pre-reflectively lived body. This is in line with the enactive perspective, which points to the importance of interactional dynamics in the constitution of individual behavior and the coordination of interpersonal behaviors (De Jaegher et al., 2010; Laroche et al., 2014).

Our design and results support those obtained with the cross-perceptual paradigm (Auvray et al., 2009), where participants interact by controlling the most minimalistic agents possible (a unique form of sensory stimulation that signals the presence of the other agent when she is within the receptive field of the participant). This paradigm first demonstrated that individual behaviors can be driven by the collective dynamics that emerge from their interaction (Auvray et al., 2009). Our method proved useful to relate these dynamics to their associated lived experiences (Froese et al., 2014b). Finally, it helped show that even the imitation of bodily behaviors can emerge from interactional dynamics, making the explicit (reflective) perception of one’s own bodily expressions unnecessary for imitating each other’s bodily behavior (Lenay and Stewart, 2012). Our preliminary results add to this line of inquiry, indicating that the disappearance of one’s own body from our perceptual field can strengthen the coupling with the other.

### Behavioral Coupling and Lived Experiences

The correlation between behavioral coupling and lived experience (as probed through the questionnaire) suggests that the strengthening of the coupling has an experiential counterpart. Indeed, in the absence of one’s own hands, the feeling of closeness was stronger when partners’ displacements had more similar velocity (as revealed by RHV). Another finding showed that participants were more likely to signal an enhanced feeling of closeness with their partner in the NO-HAND situation when the velocity of their own displacements (but not those of the partner) was lower. It thus seems that velocity information is linked to self-other integration in lived experience, whether in relational terms (velocity similarity) or in absolute value (individual velocity).

Velocity information has been shown to convey fundamental information for the recognition of animateness (Tremoulet and Feldman, 2000). It is also determinant in interpersonal movement coordination. Indeed, the mere perception of human motion velocity profiles interferes with the execution of arm movements (Kilner et al., 2007), and the perception of velocity profiles modulates our coordination with an external stimulation (Varlet et al., 2014). Hartmann et al. (2019) showed that hand movement and similarity in the frequency of movements of dancers influenced how observers perceive the degree of interaction between them. Regarding velocity attunement between partners, Słowiński et al. (2016) showed that “*dynamic similarity*” between the individuals’ spontaneous motion (according to an index derived from velocity profiles) enhances their interpersonal coordination. It thus seems that the respective velocity profiles of the spheres were important informational cues that led to more or less coordinated patterns through mutual interference and adjustments in the dynamical course of interactions.

Concerning the quality of lived experience associated with interactions, coordination has been recurrently shown to boost affective rapport and/or social connection (Marsh et al., 2009). Our results are notably consistent with those of Sun et al. (2019), who observed positive correlation between head synchrony and feelings of closeness with the partner during conversations in VR. Perceived synchrony has also been shown to increase subjective empathy (Koehne et al., 2016), while objective synchrony between behaviors correlates positively with the subjective estimation of affiliation (Hove and Risen, 2009), rapport (Miles et al., 2009) and closeness/intimacy (Sharon-David et al., 2019). A number of neuroscience studies investigating brain and behavioral activities during interpersonal coordination have guided their analysis with subjective reports (e.g.,Fairhurst et al., 2014). Kokal et al. (2011) showed that participants preferred to be paired with virtual agents that followed them and that this was sufficient to activate brain regions associated with reward processing. Cacioppo et al.’s (2014) results show that synchronized partners seem to be assimilated more, or processed as more similar to the self. Movement synchronization thus seems to enhance shared agency (Pacherie, 2012). Relatedly, movement synchrony has been shown to entail perceived entitativity (Lakens, 2010) and group-bonding (Tarr et al., 2015). Synchrony can thus elicit the feeling of belonging to the same unit. These results resonate with our observation of the correlation between velocity attunement and the feeling of closeness and point to a causal link between socio-motor and socio-affective variables. Indeed, the disappearance of one’s body increased the coupling with the movement of the other, and it is in this particular condition that we found correlations between synchrony and affiliation. It thus seems that it is the enhancement of interpersonal coordination that entailed stronger feelings of closeness, perhaps by making interacting partners’ feelings more similar (or integrated) to each other and therefore feeling more as belonging to the same unit.

Another correlational result links the slowness of a participant’s movement to their feeling of closeness to the partner. This result is consistent with Noy et al.’s (2015, Table 7) study. Using the two-dimension mirror game set-up where participants had to imitate each other’s improvised hand motion, Noy et al. (2015) found that participants reported moments of togetherness during periods of slower movement though this feeling was not reflected in the objective quantification of kinematic synchrony (co-confident motion). A possible explanation of our result is that slower movements might have allowed participants to stay more attentionally focused on, and therefore aware of and affected by, their partner’s movement. Alternatively, a feeling of closeness with the partner could have encouraged the participant to slow down, especially in the challenging NO-HAND condition, to “help”the partner follow or process one’s movement, similar to how we introduce disfluencies in conversations to help the listening partner have more “time”to process the information of our speech (Walker et al., 2014). Our result that the feeling of closeness was not correlated with the slowness of the partner argues against an explanation of the feeling of closeness as causally dependent on the partner’s slower movement (“*because you move slower I feel more connected to you*”).

Here, slowing down as a joint attention mechanism is particularly highlighted when participants don’t see their own body since their own kinesthetic “feedback”integrates the movement of the partner. Overall, we favor the hypothesis according to which slower movement might have facilitated movement coordination, which in turn strengthens socio-affective connection (with a positive valence in our experiment and in most studies available in the literature, although it can also lead to stronger connection of an opposite valence; see Di Paolo and De Jaegher, 2012; García and Di Paolo, 2018). Such a hypothesis shall be tested more specifically in future experiments, in particular with the aim of disentangling the pace of movement and the similarity of velocity, which are highly correlated in our preliminary data.

Although correlational, our results expand the literature on the links between motor and socio-affective coordination, that most often refers to rhythmical tasks, to situations where movements are free of any rhythmical constraints. They also argue in favor of the suitability of SDR for the study of relational movement and bodying, and its unique ability to create sensorimotor couplings that can help disentangle the mechanisms of intersubjective coordination. They are also coherent with the enactive perspective where the interpersonal sensorimotor coupling is at the source of experiential attunement, or the mutual incorporation of each other’s experience (Fuchs and De Jaegher, 2009). Thinking of our correlational results along with the relational approach in disability studies (Moser, 2006; Goodley, 2017) and Manning’s (2009) argument that movement creates space, we speculate that future experiments might build on our findings in searching precise articulation of the link between relational movement and intersubjective experience.

### Notes on the Method

The Tate event was a singular happening, and our challenge was to put together a prototype of an epistemological framework that takes into account the rich specificity of the event, with its inherent complexities, while allowing for quantitative observations and qualitative exploration. As a proof of concept, we gauged the capacity of our SDR set-up to provide methodological opening for a multi-perspective interrogation into the lived experiences of relational movement and bodying through dis/embodiment. We approached interrogation into the lived experiences with a self-report questionnaire which targeted particular experiences. The questionnaire was designed based on the theoretical issues at hand and using as much as possible formulations synthesized from the language used by participants in their feedback during previous sessions. One of the aims of this study was to see if SDR as a methodological device can produce meaningful experiential data.

In this paper we evaluated the link between behavioral measurements and participants’ lived experiences. The finding of significant correlations is encouraging since the behavioral measurements we used were rather general (e.g.,overall lateral head or bimanual 3D movement velocity) and did not target specific movement aspects. Plus, the mere fact that the computations were averaged over long (3 min) trials masks the singularity and the variety of the coordination patterns observed in such ill-constrained situations. This prevents us from articulating detailed movement events with particular and locally associated lived experiences. Similarly, the propositions we formulated in the questionnaire addressed lived experiences at the coarse-grain level of specific conditions in their entirety, unlike in-depth elicitation protocols and interviews used by pragmatic phenomenologists that cover seconds (Petitmengin, 2006; De Jaegher et al., 2017; Ollagnier-Beldame and Coupé, 2019). Furthermore, our participants proceeded to the questionnaire only after completing the whole experiment in which the experimental conditions were not clearly isolated in time from one another but rather passed in one continuous run. This contrasts with neurophenomenological approaches where elicitation of experience is more fine-grained and targets trials that are much shorter (e.g.,one perceptual event, see Lutz et al., 2002). In spite of these facts, the statistical significance of our results suggest that our device was successful in revealing bodying experience and creating a link between first-person testimonies and instrumental measurements. One particularly encouraging aspect of the results is that significant correlations were specific to the condition that was targeted by the question. Indeed, the feeling of closeness in absence of the vision of one’s own hands was actually significantly (and negatively) correlated with MV and RHV in that very condition, but not with these indices measured in the presence of the said vision of one’s own hands. This specificity in the correlations suggests that participants were able to discriminate and refer to the specific experimental conditions post-hoc and retrieve feelings specific to a given condition.

In our ongoing and future research, we intend to attend to the above mentioned problem of granularity, in both the behavioral and experiential domains, by endorsing a more dynamical approach. On the behavioral side, we are currently developing non-linear tools to better capture the temporal evolution of the movement variables such as new methods of recurrence quantification (Lancia and Rosenbaum, 2018). To collect more temporally fine-grained experiential reports, we plan to offer the participants the means to revisit their experiences by navigating a reconstructed video of their interaction. We can then relate specific moments of experience with the local behavioral dynamics. Moreover, this would allow us to interrogate partners about the “same”moments in time. We hope that, with more fine-grained reconstruction of the behaviors and experiences and through combining temporally aligned reports from both partners, we will be able to have deeper insights into the nature of relational movement.

Concerning the diminished aspect, we acknowledge that diminishing perceptual information in VR does not mean that nothing is added in return. The nacreous aspect of the spheres, the marble texture of the ground, and the day-ish light were all designed minimalistically but not in the sense of proposing a mere impoverishment of reality. Rather, we intended to provide a comfortable and pleasant feeling of openness as well as a conducive place for explorative encounters. The spheres also gave inspiration to participants to create patterns and metaphors and filled their imagination to build narratives about their interaction. We saw how many studies explored the impact of body appearance upon how we feel, think, and act. In that case, we can’t argue that a body made of spheres is simply a diminution of reality, though it is “minimalist” (Vuarnesson et al., 2021). This “something else” we added will remain as an open question in this paper and should be properly investigated in the future of this project.

We also would like to point out that the way we have implemented SDR in our experiment does not take into account the experiences of bodies with disabilities. We believe that this limitation not only invites us to rethink the logic of participation in the project but also opens a broader question of which knowledge counts in academic research. We recognize that our propositions on the capacities and promises of SDR for the study of bodying have been based on and oriented towards able-bodied experiences. All our participants had a good command of their bodies and did not report motor or cognitive disability experiences. We also recognize that this methodological specification is critically examined in the broader epistemological framework which we draw upon (specifically, the relational model of disability or Haraway’s “apparatus of body production”). Though a more detailed development of this point is beyond the scope of this article, we would like to mention here that in our strategy for future research we take better care to include heterogeneous body experiences.

## Conclusion

In this paper, thinking alongside Erin Manning’s concept of bodying, Latour’s (2010)“compositionist”approach to knowledge making, and literature on “body production”relational becoming (Haraway, 1988; Moser, 2006), we explored how relational movement underlies the emergence of our bodies and selves. Our working hypothesis is that the experience of our bodily self emerges in our moving relation to people and things which we encounter. We add to this the humble speculation that this process might then provide insights into the phenomena related to dis/embodiment, the experiences of body disorders, and disabilities.

The “Articulations”installation is predicated on the intuition that a task of spontaneous sensorimotor interaction coupled with SDR constitutes a coherent methodological device for this exploration. We have demonstrated that certain manipulations of the virtual space and avatars allow for the modulation of bodily and relational experiences (dis/embodiment) and the observation of the behavioral correlates of this modulation. By testing an installation where two people can freely, spontaneously, and playfully move and interact in the same space, we articulate links between the various forms of dis/embodiment and relational dynamics of movement.

The “Articulations”installation was conceived through a participatory design process, building each feature, from the looks of the virtual movement space to the different conditions to the experience measurement devices, through an iterative exchange among scientists, artists, designers and volunteers who all tested the evolving versions of the installation. This has allowed us to orient the design of the installation towards what we refer to as SDR. SDR constitutes a virtual setup where people can interact in a minimalist environment through avatars whose embodiment is deeply reduced. Such a paradigm allows to retain (or even experientially foreground) the conditions for bodying and relational movements while making kinematics of virtual bodies more easily trackable.

We activated our device at an art-science exhibition where we collected movement data along with experiential reports about experimental manipulations where various forms of dis/embodiment and environmental changes were experienced. Although we have tested our device with only a handful of dyads, which limits the scope of our conclusions, it did offer promising results. As we predicted, suppressing the feedback of the movement of the self increased the coupling with the other. We also observed meaningful links between behavioral measurements and experiential reports that depended on the absence of feedback of one’s own hands, at both the individual and the relational level. Without visual feedback of their own movement, members of dyads that had more similar velocities reported a stronger sense of proximity with their partner. This was expected since we created a situation where the visual feedback of one’s own movement exists only in the other’s movement and in the influence we have on her, which facilitates the orientation of attention toward the coupling process itself. Furthermore, in the same condition participants who moved slower (relatively to other participants) felt closer to their partners. A possible explanation of this last result is that slower movements could enhance the attention toward the partner and the intersubjective relation itself, and/or that the feeling of closeness could bring the participant to slow down as a collaborative strategy. While the causal directionality of this correlation requires further experimentation, it appears that slower movement facilitates the emergence of a relational bodying unit (or “*joint body schema*”, Soliman et al., 2015).

The results of this proof of concept show that our SDR methodological device is suitable for exploring the link between bodily lived experiences (especially relational experiences) and movements patterns (whether they refer to patterns of motion observed in individuals or in their relations). Yet, a number of improvements must be considered for further work with our installation: methodological, epistemological, and analytical. Deeper qualitative analysis of the open-ended interviews with the participants and new forms for eliciting experiential reports are needed to enrich our inferences and attend to the specific emotional and social conditions that weave into their experiences; advanced statistical methods are required to improve the detection of relational attunement beyond linear synchronicity or delayed imitation of patterns; semiotic analysis of gestures appropriate for the specific register of spontaneous sensorimotor interactions should be deployed; inviting experiences beyond those of the able-bodied.

Some directions we hope to explore in the future include: qualitative studies of relational experiences with and within SDR; exploration of how dis/embodiment induces novel gesture qualities, and communication strategies; and implications for health and dis/ability fields. More precisely, our interest in the promises of SDR in the context of disability is not about “fixing” or rehabilitating “a body”. Rather, we would like to question if relational bodying within the SDR space may inform ethico-affective understanding of dis/ability configurations. We hope that the device we have described here, and its potential for dis/embodiment could contribute to thinking of the ways in which the body is “done”and “undone”collaboratively and to displacing dis/ability from within one’s body to the relational ecological network of social and material conditions of its emergence (Moser, 2006; Ginsburg and Rapp, 2013; Shakespeare, 2014; Goodley, 2017).

## Data Availability Statement

The datasets generated for this study are available on request to the corresponding author.

## Ethics Statement

Ethical review and approval was not required for the study on human participants in accordance with the local legislation and institutional requirements. Written informed consent to participate in this study was provided by the participants or their legal guardian/next of kin.

## Author Contributions

LV, AE, and AB collected the results. JL analyzed the data. JL and AB discussed the results. All authors designed the experiment and wrote the manuscript.

## Conflict of Interest

LV was employed by company Emotic. The remaining authors declare that the research was conducted in the absence of any commercial or financial relationships that could be construed as a potential conflict of interest.

## Publisher’s Note

All claims expressed in this article are solely those of the authors and do not necessarily represent those of their affiliated organizations, or those of the publisher, the editors and the reviewers. Any product that may be evaluated in this article, or claim that may be made by its manufacturer, is not guaranteed or endorsed by the publisher.
